# A latent trait approach to measuring HIV/AIDS related stigma in healthcare professionals: application of mokken scaling technique

**DOI:** 10.1186/s12909-016-0676-3

**Published:** 2016-05-30

**Authors:** Keivan Ahmadi, Daniel D. Reidpath, Pascale Allotey, Mohamed Azmi Ahmad Hassali

**Affiliations:** School of Pharmacy, University of Lincoln, Joseph Banks Laboratories, Lincoln, Lincolnshire LN6 7DL United Kingdom; Jeffrey Cheah School of Medicine and Health Sciences, Monash University Sunway Campus, Jalan Lagoon Selatan, Bandar Sunway, 46150 Selangor DE Malaysia; Discipline of Social and Administrative Pharmacy, School of Pharmaceutical Sciences, Universiti Sains Malaysia, 11800 Penang, Malaysia

**Keywords:** HIV/AIDS, Attitude, Stigma, Mokken scale analysis, Scale, Measurement, Healthcare professionals

## Abstract

**Background:**

The attitudes of healthcare professionals towards HIV positive patients and high risk groups are central to the quality of care and therefore to the management of HIV/AIDS related stigma in health settings. Extant HIV/AIDS stigma scales that measure stigmatising attitudes towards people living with HIV/AIDS have been developed using scaling techniques such as principal component analysis. This approach has resulted in instruments that are often long. Mokken scale analysis is a nonparametric hierarchical scaling technique that can be used to develop unidimensional cumulative scales. This technique is advantageous over the other approaches; as the scales are usually shorter, while retaining acceptable psychometric properties. Moreover, Mokken scales also make no distributional assumptions about the underlying data, other than that the data are capable of being ordered by *item* and by *person*. In this study we aimed at developing a precise and concise measure of HIV/AIDS related stigma among health care professionals, using Mokken scale analysis.

**Methods:**

We carried out a cross sectional survey of healthcare students at the Monash University campuses in Malaysia and Australia. The survey consisted of demographic questions and an initial item pool of twenty five potential questions for inclusion in an HIV stigma scale.

**Results:**

We analysed the data using the *mokken* package in the R statistical environment providing a 9-item scale with high reliability, validity and acceptable psychometric properties, measuring and ranking the HIV/AIDS related stigmatising attitudes.

**Conclusion:**

Mokken scaling procedure not only produced a comprehensive hierarchical scale that could accurately order a person along HIV/AIDS stigmatising attitude, but also demonstrated a unidimensional and reliable measurement tool which could be used in future studies. The principal component analysis confirmed the accuracy of the Mokken scale analysis in correctly detecting the unidimensionality of this scale. We recommend future works to study the generalisability of this scale in a new population.

## Background

Researchers have described HIV/AIDS related stigma across populations and across domains of interpersonal interaction [1, 3, 18, 23, 24, 28]. Of the many forms of HIV/AIDS related stigma that have been described, one of forms with the greatest potential for lasting harm is the stigma by healthcare professionals towards people living with HIV/AIDS (PLWHA). The negative attitudes compromise the quality of care to PLWHA, and can affect the willingness of PLWHA to access health settings in which they are the subject of stigmatising responses from staff [6, 20, 29].

Dealing with the attitudes of [future] healthcare professionals is central to the management of this form of HIV/AIDS stigma [13, 43]. World Medical Association – declaration of Geneva – clearly lays the foundation for the non-stigmatising attitudes and behaviors expected of the healthcare professionals: “*I will NOT permit considerations of age, disease or disability, creed, ethnic origin, gender, nationality, political affiliation, race, sexual orientation, social standing or any other factor to intervene between my duty and my patient*” [42]. The management strategies to ensure non-stigmatising attitudes and behaviors may include post-qualification training or the integration of the stigmatising attitude issues in the educational curriculum of healthcare professionals – regardless of their ethnicity, nationality, race, religious beliefs, etc. – during their initial training. The relative merits of these strategies are subject to empirical investigation, and there is no reason to believe that they are not complimentary. However, whichever strategy is adopted, the measurement of change in stigmatising attitudes is a key to the assessment of the effectiveness of the intervention. There is therefore a strong case for robust measures of HIV/AIDS related stigma, developed for [future] healthcare professionals. Monash University, because of its campuses in various parts of the world, provided a good opportunity to develop a HIV/AIDS stigma scale to measure stigmatising attitudes of healthcare students of the Australian and the Malaysian campuses. The admission criteria of the health programs are identical in both campuses. Therefore, the students’ pool consisted of the individuals who deemed to have the same levels of intellectual abilities, but coming from different social and cultural backgrounds.

A number of HIV/AIDS stigma scales have been developed to measure stigmatising attitudes towards PLWHA [2, 15, 34]. The approach to scale development has tended to rely on classical test theory, and assumed that each item (question) measured the true score (level of stigma) with error for each person [32]. Good items to include in a stigma scale were selected on the basis of their pooled reliability, or in combination with Principal Component Analysis (PCA) according to their loading on a single dimension [22]. The approach makes assumption about the normality Gaussian nature of the distribution of the responses to each item.

Mokken scale analysis (MSA) takes a different approach. It is a nonparametric hierarchical scaling technique related to Guttman scaling, and falls under the umbrella of nonparametric item response theory (IRT) [32, 40]. The point of departure from classical test theory is the underlying assumption that the probability of a person responding in a particular way to an item depends on their personal latent trait (i.e., how stigmatising their attitudes towards PLWHA actually are), and on the characteristics of the item itself (i.e., how demanding or difficult an item is in terms of eliciting a negative response towards PLWHA) [9]. Thus, MSA orders people according to their probability of responding in a stigmatising manner (i.e. their latent trait) – the monotone homogeneity (MH) assumption. It also orders items according to the probability of being answered in a stigmatising manner independent of the person answering the question – the double monotonicity (DM) assumption. If the MH and DM assumptions both hold, then a Mokken scale is established that can order *people* along a latent trait of stigmatising attitudes and order the *items* in the scale on their “difficulty”. Mokken scales also make no distributional assumptions about the underlying data, other than that the data are capable of being ordered by *item* and by *person*.

The advantage of MSA is that it can be used to develop unidimensional cumulative scales that are usually shorter than scales developed using other approaches, while retaining acceptable psychometric properties [27, 32, 33]. Recently, Nyblade *et al.* hinted at the lack of brief, simple and standardised tools measuring HIV/AIDS-related stigma as one of the barriers to scaling up stigma reduction programs in health services [19]. Thus, Nyblade and her colleagues developed an 18-item measurement tool out of which five of its items meant to measure attitudes towards PLWHA [19]. Usually it takes more time and resources for creating shorter measurement tools that would retain their acceptable psychometric properties. For instance, the HIV-Knowledge Questionnaire was a 45-item measurement tool [4] that was made shorter – 18 items – for the ease of administration while retaining its psychometric properties [5]. The Brief HIV-Knowledge Questionnaire was found to be suitable for use in clinical, educational and public health settings [5]. Moreover, some of the commonly used HIV/AIDS measurement tools might have been decontextualized as the dynamic nature of HIV/AIDS stigma is under constant change [19]. AIDS Attitudes Scale (AAS), for example, was first developed in 1992, using classical test theory approach [12]; and was further validated in 1997 [10]. Since then, except for the development of an alternative form of the scale for use in general public [11], the scale has not undergone further validations. While AAS has strong psychometric properties [12], it only measures ‘empathy’ and ‘avoidance’ as the two domains of AIDS-related stigmatising attitudes. Recently developed HIV/AIDS measurement tools tend to emphasise on other domains such as HIV positive individual’s rights to fair treatment by their family members and the members of the society [19, 36].

The aim of this study was to develop a short measure of HIV/AIDS related stigma, applying Mokken scale analysis technique, for use among healthcare professionals (in training) that had sound psychometric properties.

## Methods

### Study design, participants and data collection

A cross sectional survey was carried out of healthcare students studying at Monash University campuses in Malaysia and Australia. A total of 807 students (*N = 807*) drawn from medicine and pharmacy programs responded to the survey. Sixty-two percent (62 %) of the students were female (*n = 500, mean age = 21.2, SD = 2.46*) and 38 % were male (*n = 307*, *mean age = 21.1, SD = 2.11*).

### Procedure

The respondents completed paper-based (Malaysia) or on-line (Australia) surveys that contained demographic questions and the initial item pool of potential questions for inclusion in a HIV stigma scale. The initial pool contained 25 items based on a review of the literature – removing redundant or conceptually similar questions (see Appendix 1). Each question in the pool required a response on a seven-point scale noting the degree to which a respondent agreed with the questions and statements i.e., 1- Agree strongly, to 7-Disagree strongly, or 1-Definitely NO to 7-Definitely YES. Prior to analysis all answers were recoded (to run these tests in *mokken* package answers should be numerical) such that 0 indicated the lowest level of stigmatising attitude and 6 represented the highest level. Examples of the questions in the item pool are shown in Table 1.

**Table 1:** The final 9-item HIV/AIDS stigma scale ordered from least to most difficult item

Item Nr.	Mean score^a^	Item	Item H^b^	Violation^c^	Crit^d^
1	0.86	People with HIV should NOT be bus drivers.	0.61	0	0
2	1.05	People with HIV should NOT be religious leaders.	0.53	1	39
3	1.06	People with HIV should NOT be police officers.	0.56	2	46
4	1.15	If you come to know that your friend is HIV positive, would you continue your friendship with him/her?	0.57	1	23
5	1.27	If you come to know that your colleague is HIV positive, would you continue working with him/her?	0.56	0	0
6	1.80	Would you allow your HIV positive friend to use your bathroom?	0.55	1	60
7	1.89	Would you discourage your sibling from becoming friends with an HIV/AIDS person?	0.52	1	62
8	2.66	Would you send your child to a school where one of its teachers is HIV positive?	0.55	0	0
9	4.13	A family has a right to know if a member is infected with HIV and this is more important than a family member’s right to privacy.	0.43	0	0

Note.- Loevinger’s scale coefficient H computed on the transposed Mokken scale H^T^ = 0.53; *H* = 0.54; reliability α = 0.89

^a^Mean score ranges from 0 to 6

^b^Loevinger’s scalability coefficient

^c^The summary of the number of manifest monotonicity violation(s). Some violations are too small to be relevant [17]

^d^Crit[ical] value above 80 indicates poor items that violate the assumptions of Mokken scale

### Data analysis

The MSA was conducted using the *mokken* package in the R statistical environment ([26, 37]). The approach is still relatively unusual in the literature; and a more detailed description than is usual for a *Methods* section is provided as a guide. Readers interested in even greater detail should refer to [37–39].

The analysis considers five interrelated elements: the Loevinger’s H scalability coefficient; the monotone homogeneity assumption that *people* can be monotonically ordered according to their responses to the items; the double monotonicity assumption that *items* can be monotonically ordered according to people’s responses; the reliability of the final scale (i.e., Cronbach’s alpha) [39]; and the validity of the scale including face and convergent validity [35].

Loevinger’s *H* coefficients are important in testing and constructing Mokken scales. Loevinger’s *H* coefficient is a measure of the number of Guttman errors in the data compared with the number that would be expected by chance. A Guttman error is where a person produces a paradoxical response such as endorsing a high-difficulty item while failing to endorse a low-difficulty one [8]. Guttman errors indicate that the two items do not measure the same trait. Loevinger’s *H* value equals [1 – (actual Guttman errors/predicted Guttman errors)] [8]. The scalability coefficient for each item (*H*_*i*_), item pair (*H*_*ij*_), whole scale (*H*) and transposed Mokken scale (*H*^*T*^) may range from 0 to 1 [32, 37]. The *H* coefficient for each single item as well as for the scale has to be more than 0.30 to satisfy the assumptions of Mokken scale [32]. Widely accepted rules of thumb have developed around the use of the H coefficients, such that 0.30 ≤ *H <* 0.40 indicates a weak Mokken scale; 0.40 ≤ *H <* 0.50 a moderate Mokken scale and more than 0.50 a strong Mokken scale [37]. For example, the H coefficient for the 9-item stigma scale is 0.54 – refer to Table 1 – which demonstrates a scale with items with a 46 % rate of Guttman errors, which indicates a strong Mokken scale.

### Evaluation of the MH assumption

Within a pool of items, more than one scale may be present. The mokken package for R estimates the available, possible, Mokken scales from the data using an automated item selection procedure with a default lower bound partition coefficient (c = 0.3) [37]. Although initial items in a pool are selected by researchers with an assumption that they represent a unidimensional scale, the analysis may reveal more than one scale. The automated item selection procedure uses a hierarchical clustering algorithm that partitions a set of items into potential scales that each satisfy the basic MH assumptions (ordering of people), leaving out the items with *H* coefficients less than 0.30 as unscalable [38]. The main objective of the selection procedure is to select as many good items as possible in the first Mokken scale, which supports the monotonic ordering of people. The computed Mokken scale is the collation of items that measure a single latent trait; i.e., supports a unidimensional view of the scale. A recent innovation in the selection procedure was the implementation of a genetic algorithm to improve the partitioning of the search space [38]; and it was this algorithm that was used in the present study. The monotonicity of the monotonicity homogeneity assumption was further tested using a secondary function built into the package – “*check.monotonicity”*.

### Evaluation of the DM assumption

The mokken package has equivalent functions to test the double monotonicity assumption that examine Invariant Item Ordering of *items* and Manifest Invariant Item Ordering – interested readers are referred to [38]. Manifest invariant item ordering is designed for polytomous items and orders items by their mean score, such that the selected items follow the hierarchy from the least difficult to the most difficult item [32, 37]. The manifest invariant item ordering method identifies item pairs that violate the assumptions of double monotonicity and items are successively removed from a potential scale until no significant violations remain [38]. Eventually, the double monotonicity assumptions can be visually confirmed using P-matrices function [37].

A summary statistic “Crit(ical)” is automatically generated that provides an overview of different indicators and can be used as a guide to discard the item(s) violating monotone homogeneity and/or double monotonicity assumptions [32]. Crit values greater than 80, for instance, can indicate poor items [38]. Once invariant item ordering has been established, the transposed Mokken scale coefficient (H^T^) is used to express the accuracy of the ordering of the items in the Mokken scale [33]. When 0.30 ≤ H^T^ < 0.40 occurs, it is interpreted as a weak ordering of items, 0.40 ≤ H^T^ < 0.50 is interpreted as a moderate ordering, and H^T^ > 0.50 is interpreted as a strong ordering [38].

### Validity

The construct validity of the final scale was examined in terms of both face validity and convergent validity [35]. The convergent validity was established by examining the relationship between the final scale and six independent “yes-no” questions asking about attitudes towards people living with HIV/AIDS (Appendix 2). Specifically, respondents were grouped by the number of questions to which they responded negatively towards PLWHA; resulting in 7-groups of respondents from those showing no negative attitudes (1) to those showing negative attitudes to every question (7).

## Results

The automated item selection procedure – with lowerbound partition coefficient set to 0.3 – using the genetic algorithm, initially partitioned the 25-item questionnaire into a unidimensional, 19-item Mokken scale conforming to the monotone homogeneity assumptions of the model. This step removed 6 items with low item scalability coefficients (*H*_*i*_ < 0.30). The Loevinger’s scalability coefficient for the remaining items indicated a moderate Mokken scale (*H* = 0.43) (see Appendix 3).

A further 10 items were removed from the 19-item scale because they did not support the double monotonicity (item ordering) assumptions of the model, generating a final 9-item Mokken scale. This final scale had an H coefficient indicating a strong scale (H = 0.54) with good reliability properties (Cronbach’s α = 0.89). The H^T^ coefficient further supported the notion that the scale items were monotonically ordered relative to one another and also along the latent trait (H^T^ = 0.53). The double monotonicity assumptions of the final scale were also checked by examining the P-matrices and found to be adequate [37].

The dimensionality of the scale was further tested using principal component analysis (PCA) [16]. A visual examination of the scree-plot indicated the presence of a single (unidimensional) scale. The final 9-item scale, ordering items from the least difficult item to the most difficult item is shown in Table 1.

All but one of the items (item 9) showed strong scale properties (H > .5). No serious violations occurred, and the Crit values were all within acceptable limits.

Table 2 shows the distribution of response scores, that is, the number of responses to each of the answer categories for each of the nine items of the final scale. To simplify the interpretation the response distribution is shown visually. The categorical endpoints are shown: category 1 (non-stigmatising attitude) and category 7 (highly stigmatising attitude). Responses to the intermediate categories, however, are aggregated.

**Table 2:**
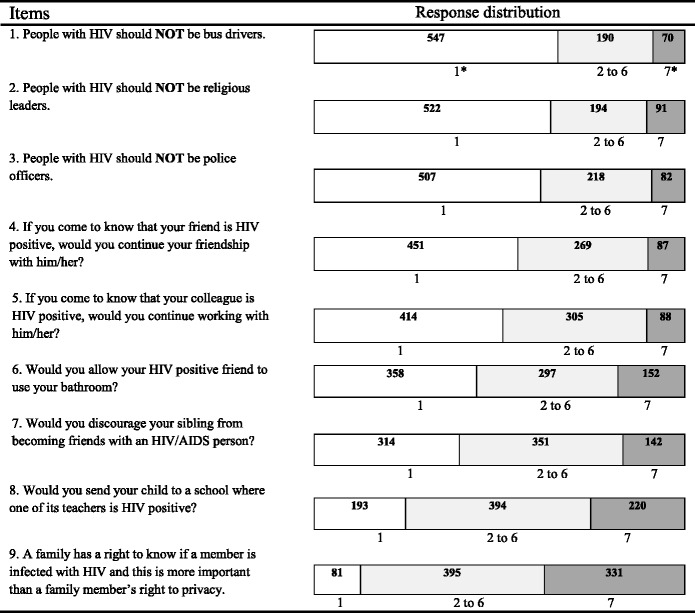
Response distribution for each of the nine items of the final scale with numbers of respondents in each category also shown

Note.- Total number of respondents (*N* = 807)

*1 non-stigmatising attitude – 7 highly stigmatising attitude

The numbers of respondents for the answer categories 2 to 6 are reported collectively

The increase in the numbers holding highly stigmatising attitudes (and concomitant decrease in numbers holding non-stigmatising attitudes) is visually, readily apparent. Sixty-eight percent (67.8 %) of respondents had no stigmatising attitudes to the idea of a bus driver with HIV/AIDS. In contrast, only 10.0 % maintained that same low level of no stigmatising attitudes when asked whether an HIV positive person was entitled to maintain their right to privacy. Using the 9 scale-items identified through the MSA, a final HIV/AIDS-stigma score was estimated for each respondent (mean = 70.6, SD = 24.34).

**Fig. 1:**
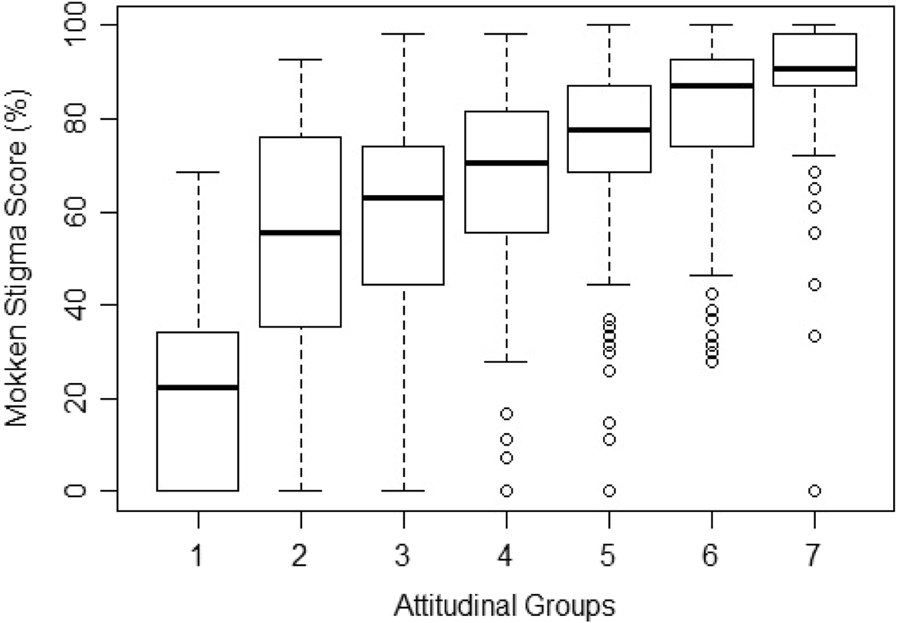
Convergent validity of the final scale

The convergent validity of the final scale was examined using a boxplot showing the distribution of stigma scores within each of the 7 attitudinal groups, where Group 7 contains individuals responding negatively to all convergent validity questions, Group 6 contains individuals responding negatively to 6 of the 7 questions, down to Group 1 containing individuals who did not respond negatively to any of the questions (Fig. 1).

The monotonically increasing, relationship between stigma score and group readily confirms the convergent validity of the scale score. The medians are strictly monotonic and increasing, as are the first quartile scores. With one small exception (Group 2) the third quartile scores are also monotonic and increasing.

## Discussion

Several scales for measuring HIV/AIDS related stigma have been developed previously [2, 15, 34]. These have relied almost exclusively on classical test theory approaches which make assumptions about the underlying normality of the distribution of responses and make no allowance for the item-trait relationship [7]. The results of the this Mokken scale analysis produced a shorter (9-item), strong (H > .5), and reliable (α = .89) scale with a logical hierarchy of item “difficulty”, and an intuitive face validity (R. [40]). The convergent validity of the scale was also established for use with students in healthcare professions.

Unlike scales derived from classical test theory, one of the appealing features of MSA (and other latent trait approaches) is that a scale has utility beyond simply providing a total item score [41]. A total score allows researchers to order people from low levels of stigma to high levels of stigma. With a Mokken scale, one can also infer relationships between items. The analysis indicated, for instance, that it was easier to be personally, socially involved with a person who is HIV positive (i.e., item 4) than it was to send one’s child to a school where a teacher was HIV positive (i.e., item 8). This graded response of items (rather than simply people) is also consistent with the framework of stigmatising responses described by which potentially provides insights into aspects of the social interaction or the kinds of social interactions that elicit more or less stigmatising responses [30].

### Limitation

There are two broad limitations associated with the analysis described here. There are some limitations on the generalisability of the findings.

The sample, was of reasonable size – certainly larger than some studies e.g.,[31] – but drawn from a single university population using convenience sampling – homogeneous at least with respect to their educational experience. Moreover, the data were collected from university students in low-prevalence settings. By virtue of this, caution should be taken when generalising the scale to healthcare professionals more broadly. However, there is some evidence to suggest that Mokken scales developed in a student population such as this are likely to generalise reasonably well to graduated healthcare professionals [25]. This, nonetheless, remains an empirical question and warrants investigation with future uses of the scale in a new population.

The other limitation, again common with scale development studies, is the possibility of maximising the scale fit to the data – over-fitting – rather than maximising the scale’s generalisability [14, 21]. To account for this limitation, we administered the 9-item Mokken scale to 352 dental students of two dental colleges. We found that except for item 6 i.e., “Would you allow your HIV positive friend to use your bathroom?” that had *Crit* value of 84, the rest of items conformed to the assumptions of Mokken scale. Moreover, the scale had a Cronbach’s alpha of 0.72. (Unpublished data).

## Conclusion

This newly developed HIV/AIDS stigma scale works well in the population of this study; however, future research could examine the generalisability of this scale in other populations such as graduated and practicing health care professionals.

## Abbreviations

AAS, AIDS attitude scale; DM, double monotonicity; HIV/AIDS, Human Immunodeficiency Virus/Acquired Immune Deficiency Syndrome; MH, monotone homogeneity; MSA, mokken scale analysis; PCA, principal component analysis; PLWHA, people living with HIV/AIDS; WMA, World Medical Association.

## Appendix 1

**Table 3:** The 25-item questionnaire aimed at measuring HIV/AIDS stigmatising attitude

Item Nr.	Item	Measure	Mokken Scale	Item H^b^
2	People with HIV should be barred from participating in contact sports like football.	HIV/AIDS stigma	1^a^	0.34
4	People with HIV/AIDS should be isolated.	HIV/AIDS stigma	1^a^	0.40
5	People with HIV should NOT be allowed to work in kindergartens.	HIV/AIDS stigma	1^a^	0.39
6	People with HIV should NOT adopt children.	HIV/AIDS stigma	1^a^	0.47
7	People with HIV should NOT be teachers.	HIV/AIDS stigma	1^a^	0.57
8	People with HIV should NOT be religious leaders.	HIV/AIDS stigma	1^a^	0.49
9	People with HIV should NOT be police officers.	HIV/AIDS stigma	1^a^	0.54
10	People with HIV should NOT be bus drivers.	HIV/AIDS stigma	1^a^	0.58
11	People with HIV should NOT be barbers.	HIV/AIDS stigma	1^a^	0.43
12	People with HIV should be allowed to travel between the countries.	HIV/AIDS stigma	1^a^	0.37
13	People with HIV/AIDS have the right NOT to reveal their status to their friends.	HIV/AIDS stigma	1^a^	0.36
14	People with HIV/AIDS have the right NOT to reveal their status to their family.	HIV/AIDS stigma	1^a^	0.32
16	A family has a right to know if a member is infected with HIV and this is more important than a family member’s right to privacy.	HIV/AIDS stigma	1^a^	0.41
17	Children with HIV in schools should be kept together in the same classroom.	HIV/AIDS stigma	1^a^	0.31
20	If you come to know that your friend is HIV positive, would you continue your friendship with him/her?	HIV/AIDS stigma	1^a^	0.46
21	Would you allow your HIV positive friend to use your bathroom?	HIV/AIDS stigma	1^a^	0.44
22	If you come to know that your colleague is HIV positive, would you continue working with him/her?	HIV/AIDS stigma	1^a^	0.46
23	Would you send your child to a school where one of its teachers is HIV positive?	HIV/AIDS stigma	1^a^	0.45
24	Would you discourage your sibling from becoming friends with an HIV/AIDS person?	HIV/AIDS stigma	1^a^	0.41
1	Governments should provide free health care to people with Type 2 diabetes.	HIV/AIDS stigma	0	0.04
3	People with HIV/AIDS should be obliged to reveal their health condition to their doctor.	HIV/AIDS stigma	0	0.17
15	People with HIV/AIDS should be penalised if they have sexual relations without revealing their health status.	HIV/AIDS stigma	0	0.11
18	Governments should provide free health care to people with HIV/AIDS.	HIV/AIDS stigma	0	0.08
19	Would you discourage your sibling from becoming friends with your close friend who has recently become HIV positive?	HIV/AIDS stigma	0	0.13
25	You are given the choice of two possible individuals as your roommate. One is a basketball player and the other one is HIV positive. Which one would you be most likely to choose?	HIV/AIDS stigma	0	0.08

^a^Loevinger’s scalability coefficient for the 19-item Mokken scale *H* = 0.43

^b^Loevinger’s scalability coefficient for each item

## Appendix 2

**Table 4:** Independent “yes-no” questions asking about attitudes towards people living with HIV/AIDS

Question Nr.	Question	Measure
1	Would you use the eating utensils of a person with HIV/AIDS?	HIV/AIDS stigma
2	Would you continue to use the services of a dentist if you learned that s/he provides dental care for patients with HIV/AIDS in her/his practice?	HIV/AIDS stigma
3	Would you sit on a toilet that has been used by a person who you learn has HIV/AIDS?	HIV/AIDS stigma
4	Would you eat in a restaurant with kitchen staff who you know have HIV/AIDS?	HIV/AIDS stigma
5	Would you be concerned if you had to have a blood test in a laboratory that provides services to a lot of people with HIV/AIDS?	HIV/AIDS stigma
6	Should a mother who has HIV/AIDS avoid physical contact with her child?	HIV/AIDS stigma

## Appendix 3

**Table 5:** The final 9-item HIV/AIDS stigma scale with answer category

Item Nr.	Item	Answer Category^a^						
		Agree strongly		Neither agree nor disagree			Disagree strongly	
1	People with HIV should NOT be bus drivers.	7	6	5	4	3	2	1
2	People with HIV should NOT be religious leaders.	7	6	5	4	3	2	1
3	People with HIV should NOT be police officers.	7	6	5	4	3	2	1
9	A family has a right to know if a member is infected with HIV and this is more important than a family member’s right to privacy.	7	6	5	4	3	2	1
		Definitely NO		Undecided			Definitely YES	
4	If you come to know that your friend is HIV positive, would you continue your friendship with him/her?	7	6	5	4	3	2	1
5	If you come to know that your colleague is HIV positive, would you continue working with him/her?	7	6	5	4	3	2	1
6	Would you allow your HIV positive friend to use your bathroom?	7	6	5	4	3	2	1
7	Would you discourage your sibling from becoming friends with an HIV/AIDS person?	7	6	5	4	3	2	1
8	Would you send your child to a school where one of its teachers is HIV positive?	7	6	5	4	3	2	1

^a^Seven point Likert scale was used for each answer category, 1 non-stigmatising attitude – 7 highly stigmatising attitude
